# Multi-Omics Analysis Provides Insights into Green Soybean in Response to Cold Stress

**DOI:** 10.3390/metabo14120687

**Published:** 2024-12-07

**Authors:** Yanhui Lin, Guangping Cao, Jing Xu, Honglin Zhu, Liqiong Tang

**Affiliations:** Hainan Key Laboratory of Crop Genetics and Breeding, Institute of Food Crops, Hainan Academy of Agricultural Sciences, Haikou 571100, China

**Keywords:** green soybean (*Glycine max* (L.) Merrill), colds stress, molecular mechanism, transcriptome, metabolome

## Abstract

Green soybean (*Glycine max* (L.) Merrill) is a highly nutritious food that is a good source of protein and fiber. However, it is sensitive to low temperatures during the growing season, and enhancing cold tolerance has become a research hotspot for breeding improvement. **Background/Objectives:** The underlying molecular mechanisms of cold tolerance in green soybean are not well understood. **Methods:** Here, a comprehensive analysis of transcriptome and metabolome was performed on a cold-tolerant cultivar treated at 10 °C for 24 h. **Results:** Compared to control groups, we identified 17,011 differentially expressed genes (DEGs) and 129 differentially expressed metabolites (DEMs). The DEGs and DEMs were further subjected to KEGG functional analysis. Finally, 11 metabolites (such as sucrose, lactose, melibiose, and dehydroascorbate) and 17 genes (such as *GOLS*, *GLA*, *UGDH*, and *ALDH*) were selected as candidates associated with cold tolerance. Notably, the identified metabolites and genes were enriched in two common pathways: ‘galactose metabolism’ and ‘ascorbate and aldarate metabolism’. **Conclusions:** The findings suggest that green soybean modulates the galactose metabolism and ascorbate and aldarate metabolism pathways to cope with cold stress. This study contributes to a deeper understanding of the complex molecular mechanisms enabling green soybeans to better avoid low-temperature damage.

## 1. Introduction

Green soybean is an immature soybean (*Glycine max* (L.) Merrill) typically harvested between the R6 and R7 stages of the reproductive cycle when the pods are green and the beans are physiologically mature [1,2,3]. It is also known as vegetable soybean, ‘mao dou’ in China, ‘edamame’ in Japan, and ‘poot kong’ in Korea [4]. Green soybean is a highly nutritious leguminous vegetable crop that contains proteins (all nine9 essential amino acids) and micronutrients (calcium, folate, iron, vitamins, etc.) [5,6]. These nutrients are integral to the human diet and help combat chronic malnutrition worldwide. Compared to grain soybeans, green soybeans have a sweeter taste and no beany flavor [4,7]. It can be boiled in salt water for appetizers and snacks or shelled and stir-fried with meat or other vegetables for curries, salads, and soups [8,9,10]. It is also an ingredient in several health foods, such as tofu and soy milk [11,12,13]. Additionally, green soybean is well suited to varieties of cropping systems due to its low input requirements, short growing season, and high soil-nitrogen-fixing capacity [14,15]. The stems and stalks of green plants can serve as fodder or green manure [16].

Global agriculture has suffered production risks and economic losses in recent years due to escalating abiotic stresses (e.g., extreme temperatures, salinity, and drought). This indicates enhancing plant resistance to abiotic stresses remains difficult [17,18,19,20,21]. Among the various abiotic stresses, cold stress is one of the most common and includes chilling stress (above 0 °C) and freezing stress (below 0 °C) [22,23,24]. Cold stress can profoundly affect plant growth, development, and productivity. The impact of cold stress on plants is multifaceted, encompassing cellular, physiological, and biochemical responses [25,26,27,28]. Green soybean cultivation in temperate climates, such as China, North America, and Argentina, tends to induce cold sensitivity when exposed to low-temperature stress [29]. Compared to unaffected plants, cold stress reduces soybean yields by an average of 24% [30]. The optimal growth temperature is 15–22 °C. Temperatures below 15 °C retard the growth of green soybean, while temperatures below 10 °C can prevent green soybean from flowering. Additionally, physiological disorders occur at temperatures below 6 °C [31,32,33]. In brief, low temperatures pose a significant barrier to green soybean growth by inhibiting metabolic and physiological activities [34,35,36]. To mitigate production losses in this scenario, there is an urgent need to breed cold-tolerant varieties. Identifying gene and metabolite changes responsive to cold stress holds great promise for accelerating the breeding of such cultivars.

High-throughput omics technologies now enable a comprehensive molecular analysis of complex biological issues, such as abiotic stress responses. Transcriptomics and metabolomics provide a comprehensive view of the molecular and metabolic changes in plants under abiotic stresses, such as cold and drought [37,38,39,40]. Transcriptomics offers insights into gene expression profiles, revealing the regulatory networks and pathways that are activated or suppressed under stress conditions. This allows researchers to identify key genes that play a role in stress conditions [40,41,42,43,44]. On the other hand, metabolomics captures the metabolic landscape of a plant and identifies the small molecules that are affected by or respond to stress [45,46,47]. These metabolites can act as stress indicators or components of a plant’s defense mechanisms. By combining these two methods, researchers gain a holistic view of how plants perceive and respond to environmental stressors. The multi-omics approach has been widely used to study abiotic stress in plants. The results have been excellent. For instance, transcriptomic analyses have identified numerous cold-responsive genes in plants, including the *GmSPS*, *B3*, *CBF*, and *MYB* genes [48,49,50,51]. Additionally, metabolomics has identified changes in metabolite profiles in cold stress-related pathways, such as soluble sugars, flavonoids, and amino acids [52,53,54,55,56]. These findings could be useful in the study of the cold tolerance of green soybean plants.

This study investigated the molecular mechanisms underlying cold stress in green soybeans using transcriptome and metabolome analyses. The aim was to identify genes, metabolites, and pathways responding to cold stress. Our findings offer new perspectives on the molecular mechanisms that underlie the adaptation of green soybeans to cold stress, potentially aiding the development of new cold-resistant cultivars.

## 2. Materials and Methods

### 2.1. Plant Material and Stress Treatment

A cold-tolerant green soybean (*Glycine max* (L.) Merrill) cultivar (Qiongxiandou15, QXD15) was utilized to investigate the defense mechanism against cold stress. QXD15 was bred by the Institute of Food Crops, Hainan Academy of Agricultural Sciences (HAAS), Haikou, China. The green soybean materials were grown under natural field conditions during normal soybean-growing seasons at the Experimental Station of the breeding base of HAAS. The materials were sown in nutrient soil in a psychrometric room at 26 °C and subjected to a photoperiod of 16 h light and 8 h dark. We selected a cold-resistant variety because it allowed us to examine the specific transcriptome and metabolome responses to cold stress without the confounding effects that might have arisen from the inherent vulnerabilities of a cold-sensitive variety. This enabled us to better understand the survival mechanisms of green soybeans under cold stress, which is critical for developing strategies to enhance cold tolerance in green soybean plants.

For the cold stress treatment, seedlings at the two-leaf-heart stage were exposed to a cold treatment of 10 °C, while an untreated group served as the control (CK). After 24 h (24 h) of cold stress exposure, leaves from five plants were collected as a sample, with three biological replicates for each sample. The samples were rapidly frozen with liquid nitrogen and kept at −80 °C for later use.

### 2.2. Transcriptome Sequencing

RNA-seq was conducted on the Illumina Novaseq 6000 platform by MetWare Biological Science and Technology Co. Ltd., following the established protocols from prior studies [57,58]. In summary, the Trizol reagent (Invitrogen, Carlsbad, CA, USA) was employed to isolate total RNA from each prepared sample. The quality of the extracted RNA was evaluated using agarose gel electrophoresis, the RNA Nano 6000 Assay Kit of a Bioanalyzer 2100 system (Agilent Technologies, Santa Clara, CA, USA), and a NanoPhotometer^®^ spectrophotometer.

### 2.3. Transcriptome Data Processing

Raw data were filtered using fastp (version: 0.19.3), primarily to eliminate adapter-contained reads. Paired reads were discarded under the following two conditions: (1) the percentage of ‘N’ bases in a read was more than 10% of the total base count or (2) the value of Q ≤ 20 bases in reads was greater than 50%. Transcriptome analysis was conducted using only the clean reads. HISAT2 (version 2.1.0) was employed for aligning the clean reads to the reference genome [59,60]. In this study, a genome of *Glycine max* was used as a reference genome (namely, Gmax_275_v2.0.fa, available at https://data.jgi.doe.gov/refine-download/phytozome?organism=Gmax&expanded=275, accessed on 10 May 2024). The new gene was predicted using StringTie v1.3.4d [61]. Transcriptome analysis was conducted based on FPKM values calculated by featureCounts v1.6.2 and StringTie v1.3.4d [61,62].

### 2.4. Metabolite Detection

The identical samples used for the transcriptome were also used for metabolomics. Metabolite detection was performed using the widely targeted metabolomics approach by the MetWare Biological Science and Technology Co. Ltd (Wuhan, China). according to standard procedures [57,63]. In summary, the prepared samples were freeze-dried and then crushed in a grinder. the After vortexing, centrifugation, and filtration, the resulting extract was kept at −80 °C for subsequent UPLC-ESI-MS/MS analysis. The metabolites were annotated by the Metware database (MWDB), and peak areas were used to quantify the abundance of metabolites [64].

### 2.5. Statistical Analysis

Principal component analysis (PCA) was performed using the prcomp function in R, with z-score normalization prior to PCA. In the clustering heatmap, Pearson correlation coefficients (PCCs) were used to represent the correlation between two samples or metabolites. The differentially expressed genes (DEGs) under cold stress were identified based on adjusted *p*-values < 0.05 and |log2 fold changes| ≥ 1 using the DESeq2 tool with the Benjamini–Hochberg method [65]. The differentially expressed metabolites (DEMs) were identified with VIP (VIP > 1) and |log2Fold Change| ≥ 1, VIP values were calculated using OPLS-DA with the R package MetaboAnalystR [66,67], and a permutation test with 200 iterations was performed to avoid overfitting. The KEGG database (http://www.kegg.jp/kegg/pathway.html, accessed on 10 May 2024) was used for gene function and metabolite annotation and pathway analysis, and the hypergeometric distribution was employed for enrichment analysis [68].

## 3. Results

### 3.1. Transcriptomic Profiling of Green Soybeans in Response to Cold Stress

After RNA sequencing, low-quality reads were removed from the raw data. The clean base for each sample exceeded 6 Gb, with GC content values greater than 44%. Additionally, Q20 and Q30 values for each were above 97% and 91%, respectively (Table S1). A genome of *Glycine max* was selected as the reference genome. The reads mapped values for both the treatment and control groups exceeded 95%. Finally, a total of 32,463 functional genes were identified and the FPKM value was calculated as the level of gene expression (Table S2). Subsequently, principal component analysis (PCA) was conducted to elucidate the temporal transnational changes between the two groups. PC1 and PC2 explained 62.81% and 13.91% of the total variance, respectively (Figure S1). PCA separated all samples into the cold treatment and control groups, with significant expression differences between the two groups, indicating that the expression of genes in green soybeans is significantly influenced by cold stress.

### 3.2. Analysis of Differentially Expressed Genes

**Figure 1:**
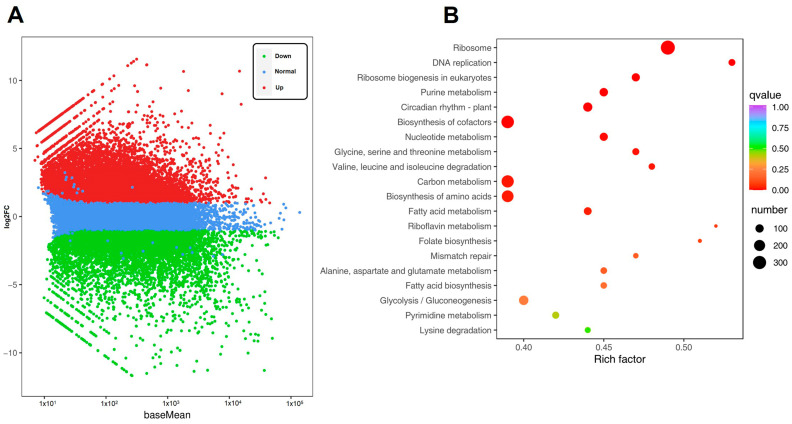
Transcriptome analysis of green soybeans (QXD15). (**A**) MA plot of the DEGs in response to cold stress. (**B**) Functional Kyoto Encyclopedia of Genes and Genomes (KEGG) pathway classification of DEGs (top 20 listed).

To explore the genetic responses of green soybeans to cold stress, we identified 17,011 DEGs comprising 9145 upregulated and 7866 downregulated genes (Table S3). The distribution of gene expression and log2FC values for these DEGs was shown in an M-versus-A (MA) plot (Figure 1A). The results showed that low temperatures could induce a number of gene expression alterations in green soybeans, thereby demonstrating active adaptation in response to cold stress.

To investigate the functions of DEGs under cold stress, all DEGs were mapped to the KEGG enrichment analysis and categorized into 137 KEGG pathways (Table S3). The most abundant terms were metabolic and genetic information processing-related pathways, including ‘Ribosome’, ‘DNA replication’, ‘Ribosome biogenesis in eukaryotes’, ‘Purine metabolism’, ‘Circadian rhythm-plant’, ‘Cofactor biosynthesis’, ‘Metabolic pathways’, and ‘Fatty acid metabolism’ (Figure 1B). The results indicated that these pathways were crucial in the response of green soybeans to cold stress.

### 3.3. Metabolic Profiling of Green Soybeans in Response to Cold Stress

**Figure 2:**
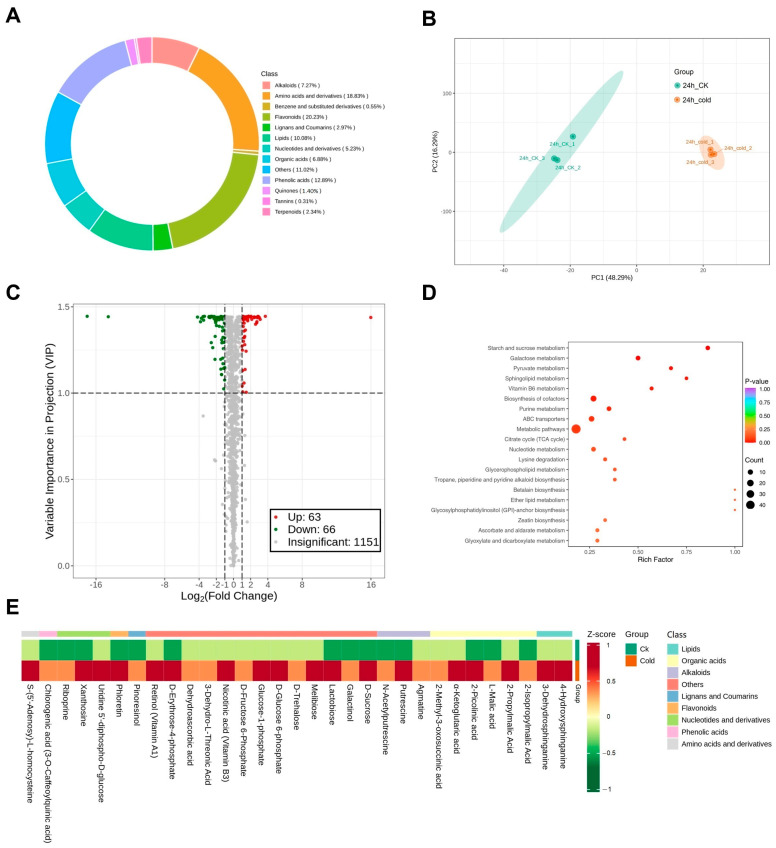
Metabolomics analysis of green soybeans (QXD15). (**A**) Classification of metabolites. (**B**) PCA of metabolites. (**C**) Volcano plot of the DEMs in response to cold stress. (**D**) KEGG pathway classification of DEMs (top 20 listed). (**E**) Heatmap of 31 DAMs under cold stress. Scaled values of the relative contents of metabolites were used for z-scale normalization.

To explore the metabolic changes in green soybeans during cold stress, a widely targeted metabolome approach was performed on the identical samples that were previously used for the transcriptome analysis. We identified and quantified a total of 1280 metabolites, comprising 259 flavonoids (20.23%), 241 amino acids and derivatives (18.83%), 165 phenolic acids (12.89%), 129 lipids (10.08%), 93 alkaloids (7.27%), 88 organic acids (6.88%), 67 nucleotides and derivatives (5.23%), 38 lignans and coumarins (2.97%), 30 terpenoids (2.34%), 18 quinones (1.41%), 7 benzene and substituted derivatives (0.55%), 4 tannins (0.31%), and 141 metabolites belonging to other chemical classes (11.02%) (Figure 2A and Table S5).

PCA was performed for metabolites. PC1 and PC2 accounted for 48.29% and 16.29% of the total variance, respectively (Figure 2B). A clear separation between the treatment and control groups was observed, with the three biological replicates in each group clustering closely together. Additionally, the cluster heatmap of the metabolites highlighted the similarity among replicates within the same group and the significant differences between the two groups. (Figure S2). The results revealed that cold stress induced significant metabolic changes in green soybeans in the two groups and that this study was reliable and reproducible.

### 3.4. Analysis of Differentially Expressed Metabolites

A total of 129 DEMs were identified between the two groups, comprising 63 upregulated and 66 downregulated metabolites (Figure 2C). All DEMs could be grouped into 11 classes, primarily including amino acids, flavonoids, organic acids, lipids, alkaloids, nucleotides, lignans, and coumarins (Table S6). We subsequently conducted a KEGG enrichment analysis to determine the functions of the DEMs. Out of 129 DEMs, 57 were annotated and classified into 72 KEGG pathways (Table S7). These pathways were mainly associated with metabolic processes, such as ‘Galactose metabolism’ and ‘Starch and sucrose metabolism’ (Figure 2D). Among the 57 DEMs, 31 exhibited significant accumulation (DAMs), and these 31 DAMs were therefore selected as candidate metabolites of cold tolerance for further integrated analysis (Figure 2E). These DAMs were mainly enriched in pathways including ‘Galactose metabolism’, ‘Ascorbate metabolism’, ‘Glycerolipid metabolism’, and ‘Flavonoid biosynthesis’.

### 3.5. Integrated Analysis of Transcriptomic and Metabolomic Data

To identify the key pathways related to cold tolerance in green soybean plants, a conjoint KEGG enrichment analysis was conducted on transcriptome and metabolome data. Additionally, we conducted a detailed examination of all conjoint pathways, excluding those where the DAMs and DEGs showed low correlation, meaning their positions in the pathway maps were relatively distant. Finally, two common enriched pathways, ‘galactose metabolism’ and ‘ascorbate metabolism’, were identified as key pathways responding to cold stress in green soybeans. These two pathways were selected for further detailed analysis due to their high enrichment, correlation, and relevance to the cold stress response.

**Figure 3:**
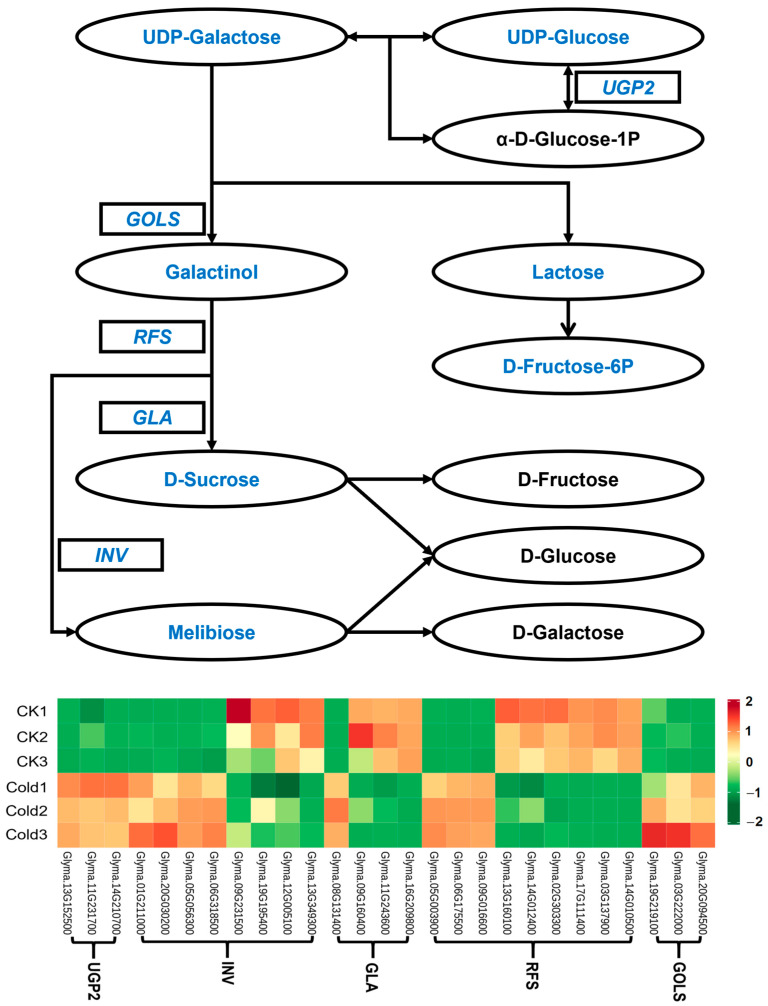
The genes and metabolites identified in the galactose metabolism pathway in response to cold stress. Blue represents the metabolites or genes that changed under cold stress. *GOLS*: inositol 3-alpha-galactosyltransferase [EC:2.4.1.123]; *RFS*: raffinose synthase [EC:2.4.1.82]; *GLA*: alpha-galactosidase [EC:3.2.1.22]; *INV*: beta-fructofuranosidase [EC:3.2.1.26]; *UGP2:* UTP-glucose-1-phosphate uridylyltransferase [EC:2.7.7.9].

In galactose metabolism, we identified seven DAMs such as sucrose, galactinol, lactobiose, and melibiose (Figure 3). Some of these DAMs were known osmotic protectants. These DAMs accumulate in green soybeans to help maintain osmotic balance and protect cellular structures from damage under cold stress. Furthermore, we observed that the levels of gene coding for structural enzymes were significantly increased under cold stress conditions. For example, cold stress upregulated three genes coding for galactinol synthases (*GOLS*, inositol 3-alpha-galactosyltransferase [EC:2.4.1.123]). We also discovered that the expressions of *RFS*, *GLA*, *UGP2*, and *INV* were induced by cold stress. Collectively, the results suggested that galactose metabolism may play an important role in protecting green soybeans against cold stress. The upregulation of genes in the galactose metabolic pathway, along with the accumulation of metabolites, work together in the cold stress response mechanism of green soybeans, helping green soybeans adapt and resist the effects of low-temperature environments.

**Figure 4:**
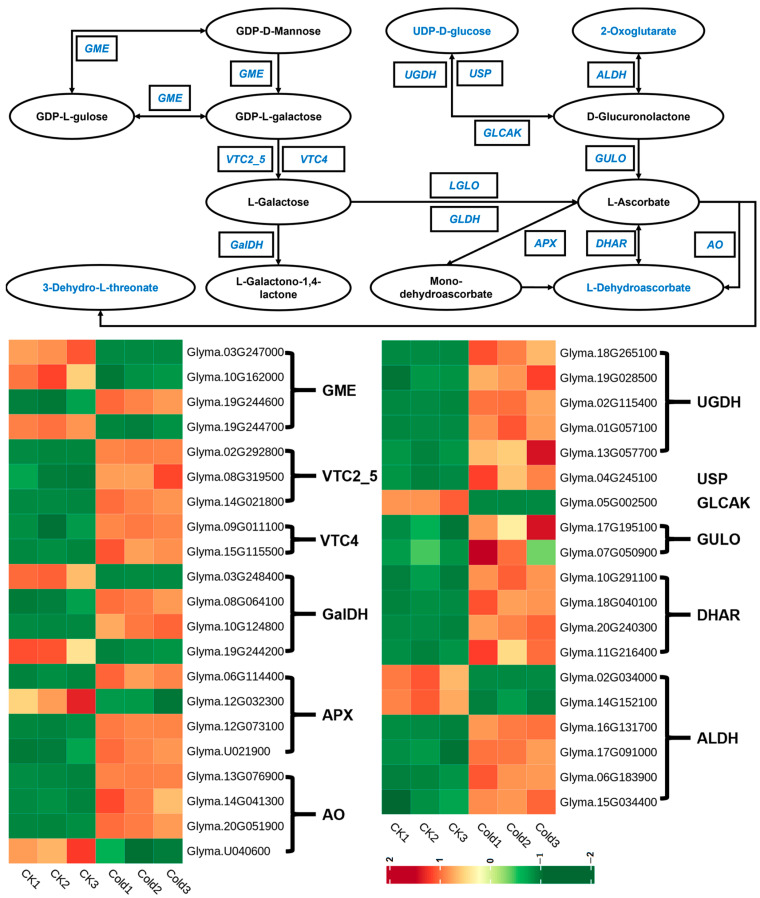
The genes and metabolites identified in the ascorbate and aldarate metabolism pathways in response to cold stress. Blue represents the metabolites or genes that changed under cold stress. *UGDH*: UDPglucose 6-dehydrogenase [EC:1.1.1.22]; *USP*: UDP-sugar pyrophosphorylase [EC:2.7.7.64]; *GLCAK*: glucuronokinase [EC:2.7.1.43]; *GULO*: L-gulonolactone oxidase [EC:1.1.3.8]; DHAR: glutathione dehydrogenase/transferase [EC:1.8.5.1 2.5.1.18]; ALDH: aldehyde dehydrogenase (NAD+) [EC:1.2.1.3]; *GME*: GDP-D-mannose 3′, 5′-epimerase [EC:5.1.3.18 5.1.3.-]; *VTC2_5*: GDP-L-galactose phosphorylase [EC:2.7.7.69]; *VTC4*: inositol-phosphate phosphatase/L-galactose 1-phosphate phosphatase [EC:3.1.3.25 3.1.3.93]; *GalDH*: L-galactose dehydrogenase [EC:1.1.1.316]; *APX*: L-ascorbate peroxidase [EC:1.11.1.11]; *AO*: L-ascorbate oxidase [EC:1.10.3.3].

Likewise, four DAMs and 12 DEGs associated with ascorbate and aldarate metabolism exhibited significantly higher levels under cold stress compared to the control group (Figure 4). For instance, L-dehydroascorbic acid and 3-dehydro-L-threonate, which were metabolites downstream in the ascorbate metabolic pathway, were observed to accumulate significantly under cold stress. Moreover, 2-oxoglutarate and UDP-D-glucose were also much higher under cold stress than in the control group. The DEGs such as UDPglucose 6-dehydrogenase (*UGDH*, EC: 1.1.1.22), UDP-sugar pyrophosphorylase (*USP*, EC: 2.7.7.64), and glucuronokinase (*GLCAK*, EC: 2.7.1.43) were upregulated. The findings indicated that the DAMs and DEGs associated with ascorbate and aldarate metabolism were taken conjointly to respond to cold stress in green soybeans.

## 4. Discussion

Climate change’s effect on crop yields is increasingly recognized due to more frequent extreme weather. Plants have developed ways to adjust gene expression and metabolism to cope with cold stress over time. Identifying cold-responsive genes and metabolites is crucial for developing cold-tolerant crops [69,70,71].

### 4.1. The Crucial Role of Galactose Metabolism Under Cold Stress in Green Soybean

Low temperatures can result in the accumulation of reactive oxygen species (ROS) and protein denaturation in plants [38,72,73]. Soluble sugars act as osmotic protectants to mitigate the effects of cold stress through osmotic adjustment [74,75]. The accumulation of these sugars can enhance cell water potential, increase cell water holding capacity, and reduce the cytoplasmic freezing point [76]. Soluble sugars are also an essential energy source for plants during cold stress [23,77]. These sugars are particularly important when photosynthesis is reduced due to low temperatures, maintaining basic metabolic processes and enhancing the plant’s ability to withstand cold conditions. Therefore, the cold tolerance of plants is directly related to changes in these sugar levels under cold stress. In this study, the levels of soluble sugars, such as sucrose and trehalose, were greatly increased under cold stress compared to the control group (Figure 2E and Figure 3). These results are consistent with previous studies, which found higher levels of trehalose in the cold-stressed rice and higher levels of galactose and sucrose in the cold-stressed wild strawberry and peach [57,78,79]. It is notable that the metabolites associated with osmotic adjustment, including proline, glucose, and fructose, were not found to accumulate significantly. However, we found an increase in the concentrations of glucose and fructose derivatives, including glucose-1-phosphate, D-glucose-6-phosphate, and D-fructose-6-phosphate. Additionally, the content of phloretin increased, which acts as an inhibitor of glucose cotransporters. Therefore, we hypothesize that low-temperature stress triggers the catabolism of existing glucose to supply energy to the plant, while the increased phloretin restricts the synthesis of new glucose, thereby preventing an increase in glucose content.

Additionally, several studies have shown that some plants can enhance tolerance to abiotic stress by synthesizing protective metabolites, such as galactinol. Galactinol can act as an osmotic protectant, antioxidant, and signaling molecule [80,81,82]. As the temperature drops, the accumulation of galactinol increases the solute concentration inside the cells. This, in turn, lowers the water potential of the cells, enabling the plant to absorb and retain water more effectively. For example, in some cold-tolerant plant species, a significant increase in galactinol levels has been observed during cold periods, which is associated with enhanced water retention and reduced dehydration damage. Galactinol has antioxidant properties. Low-temperature stress often leads to the over-production of reactive oxygen species (ROS) in plants, such as superoxide anions (O_2_^−^), hydrogen peroxide (H_2_O_2_), and hydroxyl radicals (·OH). Galactinol can scavenge these ROS, thereby reducing oxidative damage to plant cells. It can react with ROS and convert them into less harmful substances. This antioxidant activity helps to protect plant membranes, proteins, and nucleic acids from oxidative degradation, which is crucial for maintaining normal cell function during cold stress. In addition, changes in galactinol levels can initiate a series of signaling events within the plant. For example, it might interact with specific receptors or proteins involved in cold-sensing and signal-transduction pathways. This interaction can subsequently result in the upregulation of genes that encode cold-responsive proteins, such as dehydrins and late embryogenesis abundant (LEA) proteins, which are crucial for shielding the plant against cold-induced damage. In this study, galactinol was also specifically accumulated under cold stress.

### 4.2. The Crucial Role of Ascorbate and Aldarate Metabolism Under Cold Stress in Green Soybeans

As noted above, low temperatures can induce the accumulation of *ROS* in plant cells, resulting in cell apoptosis and metabolic disorders. To survive, plants scavenge excess ROS with antioxidants such as ascorbate. Ascorbic acid is a potent antioxidant that protects cells from damage by free radicals [83,84,85]. Previous studies showed that ascorbic acid is regenerated in plants through the ascorbate–glutathione cycle (AsA-GSH cycle) [86,87,88]. In this cycle, ascorbate acts as an electron donor for ascorbate peroxidase (*APX*), scavenging H_2_O_2_, and is itself oxidized to monodehydroascorbate (MDHA), which is further converted to dehydroascorbate (DHA) and finally regenerated to ascorbic acid using glutathione (*GSH*) as a reducing agent, catalyzed by dehydroascorbate reductase (*DHAR*) (Figure 4). In this study, the content of ascorbate was not found to increase, but the level of its downstream product, dehydroascorbate, increased significantly. Meanwhile, *DHAR* was found to accumulate significantly. It is suggested that ascorbate was actively functioning as an antioxidant, scavenging excess *ROS*.

Glutathione is a tripeptide composed of glutamate, cysteine, and glycine, and it plays a crucial role in cold stress [89,90,91]. Cold stress can induce the production of reactive oxygen species (ROS), which can cause oxidative damage to cellular components, including proteins, lipids, and DNA. Glutathione acts as a critical antioxidant by directly scavenging ROS, thereby reducing oxidative stress and protecting plant cells. Glutathione is also involved in the regeneration of other important antioxidants, such as ascorbate (vitamin C) and tocopherols (vitamin E). It helps maintain a balance in the antioxidant defense system by serving as a reducing agent, thus enhancing the overall capacity of the plant to cope with oxidative damage. *GSH* is a co-factor for several antioxidant enzymes, including glutathione peroxidases and glutathione S-transferases, which play specific roles in detoxifying peroxides and conjugating harmful compounds, respectively. In this study, *GSH* was involved in ascorbic acid metabolism, which enhances cold resistance in green soybean plants. Additionally, *GSH* can also act as a signaling molecule, influencing the activation of various cellular pathways in response to stress, including those involved in cold stress adaptation. GSH is involved in cellular signaling pathways that regulate various stress-responsive genes. It modulates the expression of genes related to stress tolerance and metabolic pathways during cold stress, ensuring that plants can adapt appropriately. Glutathione can be enzymatically converted to hydrogen sulfide (H2S), a signaling molecule involved in various physiological processes in plants. Under cold stress, H2S production can enhance stress tolerance by promoting stomatal closure, regulating ion homeostasis, and triggering the synthesis of protective compounds. Glutathione maintains cellular redox homeostasis, acting as a redox buffer. Changes in the *GSH*/*GSSG* (oxidized glutathione) ratio can serve as a signal for the onset of stress responses and influence the expression of stress-related genes and proteins [92]. In soybeans, cold stress can lead to reduced photosynthesis, impaired growth, and developmental delays. The presence of adequate levels of glutathione helps mitigate these effects by promoting the repair of damaged molecules, maintaining metabolic functions, and supporting overall plant resilience. Glutathione can stimulate the production of secondary metabolites, such as flavonoids and phenolics, which not only contribute to antioxidant defense but also play roles in stress signaling and plant defense mechanisms. In this work, several DAMs, such as phloretin and chlorogenic acid, were enriched in the flavonoid biosynthesis pathway. We hypothesize that *GHS* may be involved in this process and may play a significant role. Phloretin and chlorogenic acid are both approved antioxidants. *GSH* plays a critical role in green soybeans under cold stress, functioning as both an antioxidant and a signaling molecule.

## 5. Conclusions

In this study, a comprehensive analysis of transcriptome and metabolome data was conducted to reveal the molecular response to cold stress of green soybeans. This study successfully identified novel regulatory and functional candidates involved in galactose and ascorbate metabolisms that contribute to cold tolerance in green soybeans. The findings suggest that green soybeans modulate the galactose metabolism and ascorbate and aldarate metabolism pathways to enhance energy storage, maintain osmotic balance, and bolster antioxidant defense systems in response to cold stress. Lastly, the results of this study can not only deepen our understanding of the cold stress response mechanisms of green soybeans but also provide potential molecular markers and genetic resources for the development of more cold-tolerant crop cultivars.

## Data Availability

The raw sequence data reported in this study have been deposited in the Genome Sequence Archive [93]. National Genomics Data Center [94], China National Center Bioinformation/Beijing Institute of Genomics, Chinese Academy of Sciences (GSA: CRA019297) that are publicly accessible at https://ngdc.cncb.ac.cn/gsa/ (accessed on 2 October 2024).

## Supplementary Materials

The following supporting information can be downloaded at https://www.mdpi.com/article/10.3390/metabo14120687/s1: Figure S1: PCA of gene expression; Figure S2: Heatmap of DEMs; Table S1: Summary of the RNA-seq data collected from green soybean (QXD15) samples; Table S2: Gene expression profiles of green soybean (QXD15) samples treated at 10 °C for 24 h; Table S3: Differentially expressed genes in the comparison of cold treatment and control groups; Table S4: KEGG pathways annotated by DEGs; Table S5: List of metabolites detected in the green soybean samples; Table S6: Differentially expressed metabolites in the comparison of cold treatment and control groups; Table S7: KEGG pathways annotated by DEMs.
