# Supplementary figures and images for: Multi-Omics Analysis Provides Insights into Green Soybean in Response to Cold Stress
Source: Metabolites. 2024 Dec 7;14(12):687. doi: 10.3390/metabo14120687 (PMC11678371; doi:10.3390/metabo14120687)

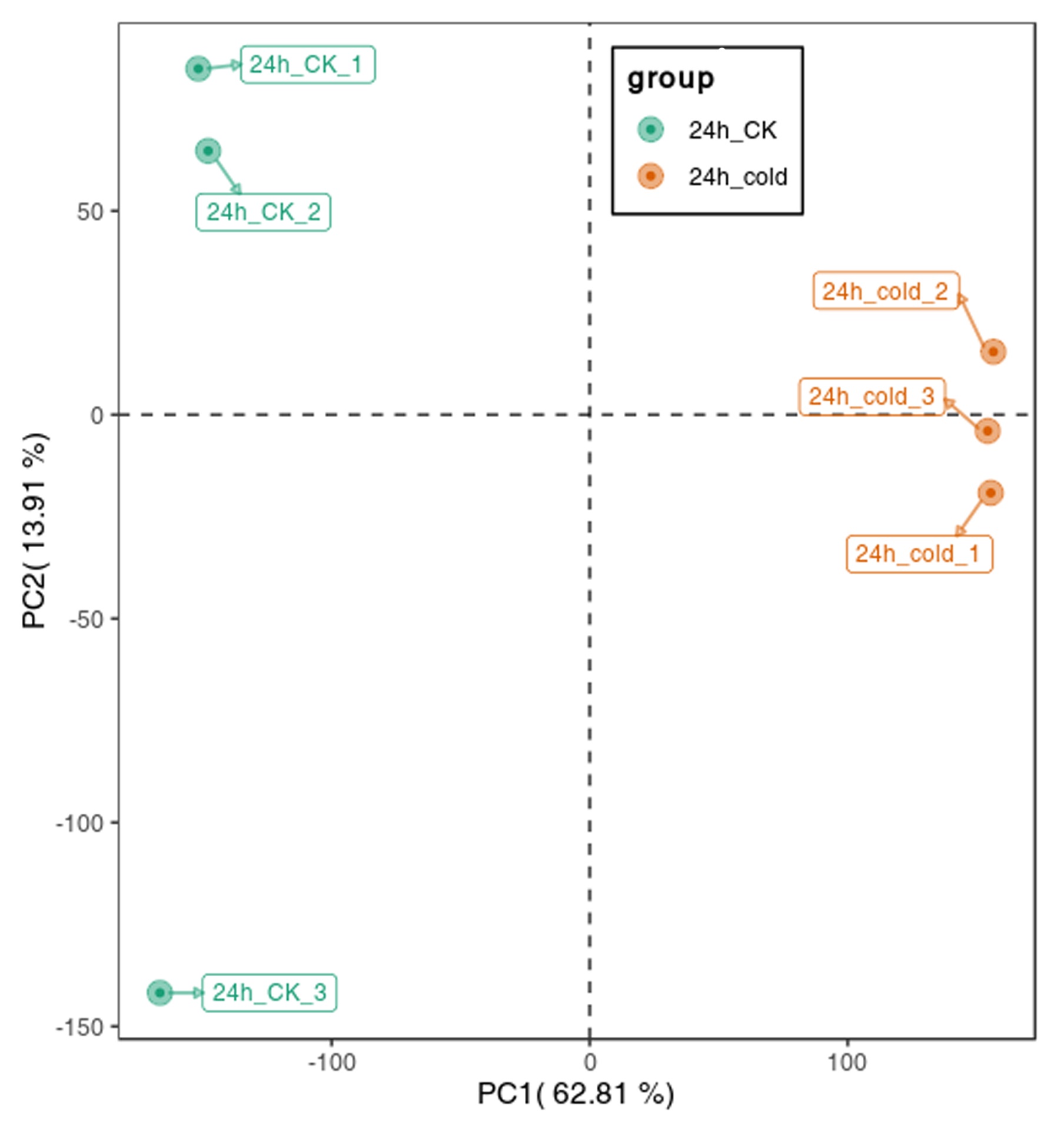

Supplement: Supplementary file 1 [file metabolites-14-00687-s001.zip › Supplementary Figure S1 PCA of gene expression.jpg]

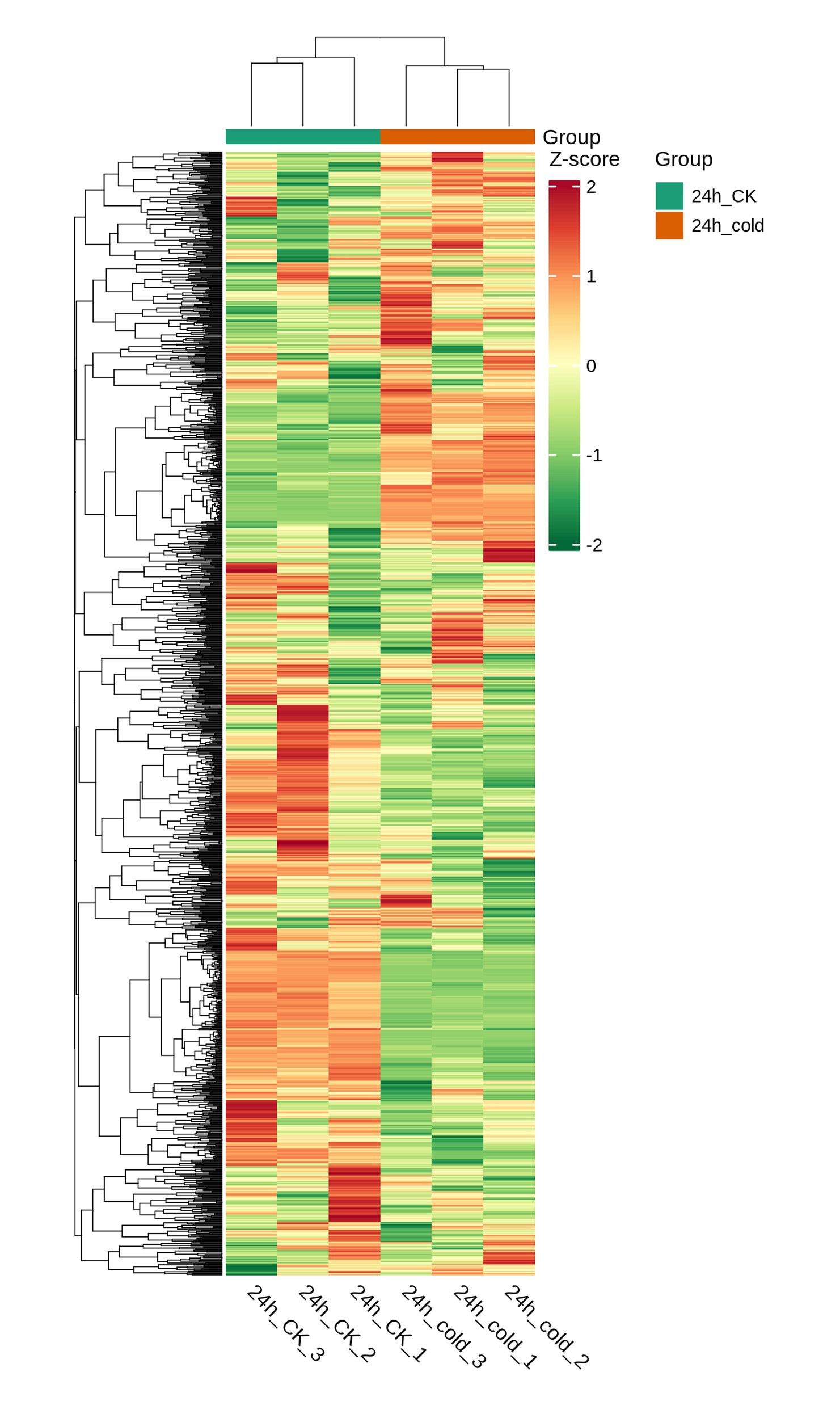

Supplement: Supplementary file 1 [file metabolites-14-00687-s001.zip › Supplementary Figure S2 Heatmap of DEMs.jpg]
